# Impact of ENT resource nurses in improving operating room efficiency

**DOI:** 10.1186/s40463-020-00431-8

**Published:** 2020-07-23

**Authors:** Kaishan Aravinthan, Connor Holmes, Sreejit P. Nair, Anil R. Sharma, Russell A. Murphy

**Affiliations:** Division of Otolaryngology - Head and Neck Surgery, Department of Surgery, College of Medicine, University of Saskatchewan, Saskatoon, Saskatchewan S7N 5E5 Canada

**Keywords:** ENT resource nurse, OR efficiency, ENT surgeries

## Abstract

**Background:**

Operating room (OR) efficiency is related to minutes spared from surgical time and has been linked to the make up of surgical teams and operating room workplace. The research on the efficiency of surgical nursing staff members is scant. The current study evaluates the effect of ENT trained OR resource nurses on the efficiency of operating time during ENT procedures.

**Methods:**

Five hundred seventy-three ENT surgery cases from 4 surgeons were retrospectively reviewed. Two hundred forty-two cases had ENT OR nursing staff and 331 cases had non-ENT OR nursing staff. Requested operative times (ROT) and true operative times (TOT) were analyzed. The difference between the TOT and ROT was used to measure operating time efficiency.

**Results:**

Cases with ROT < 30 min (M = -1.19, SD = 5.01) required 3.34 min less than planned for when an ENT nurse was present compared to those with non-ENT nursing staff which required on average 2.15 min (M = 2.15, SD = 5.68) longer than ROT. Furthermore, cases with ROT > 30 min (M = -4.32, SD = 10.85) required 10.85 min less than planned for when an ENT nurse was present. Conversely with non-ENT nursing staff cases with a ROT > 30 min required on average 6.53 min (M = 6.53, SD = 11.85) longer than ROT.

**Conclusion:**

ENT resource nurses were shown to improve OR efficiency in cases less than 30 min and greater than 30 min. Cases that were greater than 30 min showed the largest increase in efficiency. Specialized ENT nursing staff improved efficiency during common ENT surgeries.

**Graphical abstract:**

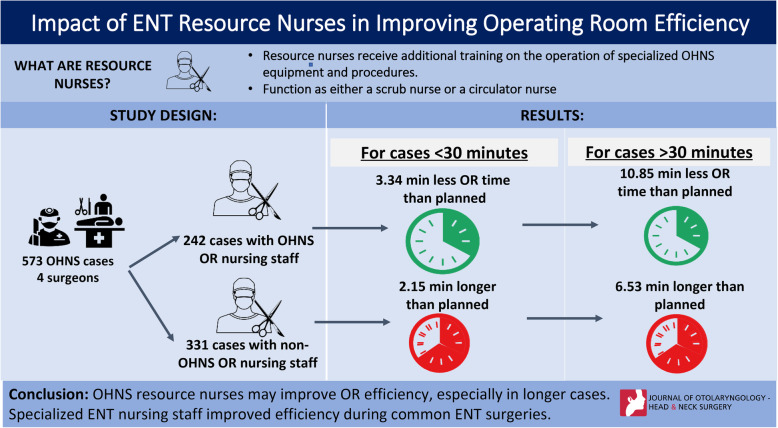

## Background

Operating room time availability is an issue both provincially and nationally. Operating rooms (OR) are a relatively fixed asset, however the need for operating room time continues to grow. This discrepancy results in the healthcare system being unable to keep up with the demand for operating time [1]. A greater focus needs to be placed on utilizing existing operating time efficiently [2–4]. Surgical procedures are variable in their resource requirements. Required time, nurses, anesthesiologists, surgeons, and equipment are a few of the many variables, that change from procedure to procedure. This variability can make it difficult to maximize the efficiency of each procedure [5].

The specific requirements and experiences of anaesthesia and emergency patients lead to the development of the specialist nursing role, who care for patients undergoing surgery or invasive procedures [6, 7]. As part of the perioperative team, they can collaborate with surgeons and anesthesiologists to facilitate surgical procedures, to improve surgical (clinical) outcomes and to enhance patient satisfaction [8]. In Saskatoon, ENT surgeons have access to specialized ENT resource nurses. The resource nurses receive additional training on the operation of specialized ENT equipment (CO_2_ Laser, Navigation Systems etc.) and specific resource requirements for various ENT procedures. ENT resource nurses can function as either a scrub nurse or a circulator nurse within the operating room. Their experience and additional training make them a valuable part of the ENT operative team.

We are interested in evaluating the effect of ENT resource nurses on improving operative efficiency. The current study utilizes the retrospectively collected data on the time, used by various surgeons in the operating room during different scheduling time points, to calculate the differences among them using inferential statistical analysis.

## Methods

### Experimental design of the study

A retrospective review of Saskatchewan Health Authority (SHA) electronic surgical data was conducted after obtaining approval from the University of Saskatchewan research ethics board. The surgical data of four staff ENT surgeons was sampled. The inclusion criteria was provided to an SHA data analyst, who was blinded to the project objective. The SHA data analyst provided the research team with deidentified raw data from the electronic surgical database.

### Inclusion/exclusion criteria

A total of 649 procedures completed between January 1st and March 31st of 2018 were sampled. All procedures were carried out at the same location – a tertiary care public hospital (St. Paul’s Hospital, Saskatoon). Procedures included were; septoplasty, rhinoplasty, laryngectomy, CO_2_ laser throat surgery, tonsillectomy with or without adenoidectomy, sinus surgery (image guided or non image guided), thyroidectomy (partial or full), parathyroidectomy, myringotomy, tympanoplasty parotid gland surgery, and sub-mandibular gland surgery. Fourty-Nine cases were removed due to delays unrelated to the study outcomes (patient arriving late, staff arriving late, PACU full, anaesthesia technical difficulties, surgical complication, trauma and/or staff shortage). Twenty-five cases were identified as emergent and hence removed from the study. Two cases were identified as outliers as a result of a data-entry error and removed. Thus, a total of 573 cases were identified for statistical analysis. A total of 331 cases did not had an ENT resource nurse participation, while 242 cases did have an ENT resource nurse.

#### Primary and secondary test groups

Cases were divided into four primary groups and four secondary groups. The primary test groups were: Group A (Cases without an ENT Resource Nurse); Group B (Cases with an ENT Resource Nurse); Group I (Cases with ROT < 30 mins); Group II (Cases with ROT > 30 mins). The primary test groups are illustrated in Table 1. The secondary test groups were divided based on the requested operative time (ROT) and ENT resource nurse participation. The secondary test groups are illustrated in Table 2. The ROT represented the total procedure length anticipated by the surgeon at the time of case booking. The ROT was used to split up minor procedures (bilateral myringotomy and tube placement, tonsillectomy, adenoidectomy, etc) from major procedures (Parotidectomy, thyroidectomy, etc).

**Table 1:** Primary test groups. Number of cases (N) distributed per group used in the study based on the ENT resource nurse participation and case length

Groups	N
Group A (Cases without an ENT Resource Nurse)	331 Cases
Group B (Cases with an ENT Resource Nurse)	242 Cases
Group I (Cases with ROT < 30 min)	258 Cases
Group II (Cases with ROT > 30 min)	314 Cases

**Table 2:** Secondary test groups. Number of cases (N) distributed per group used in the study based on the ENT resource nurse participation and requested operative time (ROT). The ROT of < 30 min and > 30 min were used to categorize minor and major procedures respectively based on the anticipated total procedure length

Group	N
Group 1 (Cases without an ENT Resource Nurse and ROT < 30 min)	144 Cases
Group 2 (Cases with an ENT Resource Nurse and ROT < 30 min)	114 Cases
Group 3 (Cases without an ENT Resource Nurse and ROT > 30 min)	187 Cases
Group 4 (Cases with an ENT Resource Nurse and ROT > 30 min)	127 Cases

#### Outcome measures

**Fig. 1:**
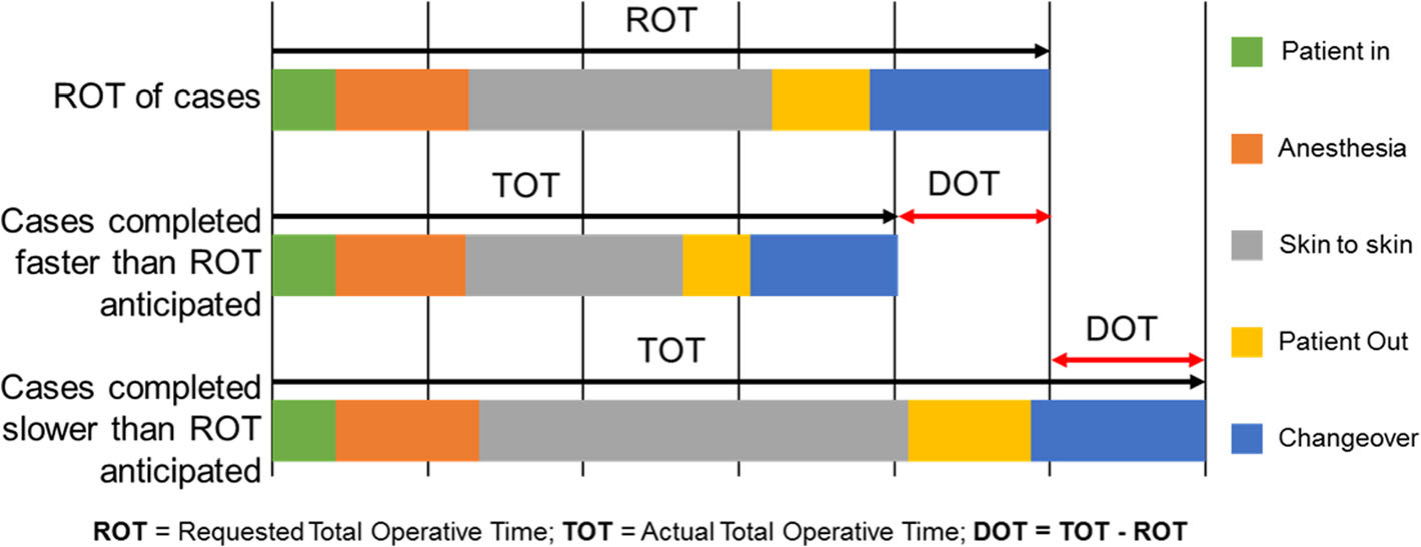
End points used. Schematic representation of the various end points used in the study

For each case the following times were recorded by OR staff: ROT, scheduled start time, patient in room, patient out of room, total anesthesia time, skin to skin time, delay duration, delay reason and OR nurse. Based on the recorded times the following time intervals were calculated: total operative time (TOT) and difference in operative time (DOT). The TOT represents the total amount of anesthesia time, skin to skin time and changeover required for a case. The ROT represents the TOT predicted by the surgeon when booking the case. The ROT also accounts for unique patient considerations made by the surgeon, while booking the case. Two patients requiring the same procedure may have different ROT’s, based on presentation and anatomical challenges identified by the surgeon. Additionaly, the ROT considers the time the surgeon anticipates they need to complete the procedure based on their experience level. The DOT represents the difference between the actual total operative time taken (TOT) versus what was initially requested (ROT). A positive DOT represents a case that took longer than planned for, while a negative DOT illustrates a case completed faster than planned for. Figure 1 provides a schematic illustrating the calculated end points discussed above.

The data samples were further grouped based on the scheduling order of the procedure carried out in the OR. The cases were classified as – first case (FC), in between cases (IBC) and last case (LC). The variance among the DOT between different classified schedueling order classes was also calculated.

### Statistical analysis

Prior to data analysis, all the data was de-identified, and then logged into SPSS 25 for analysis. For all analytics, an alpha value of 0.05 and 0.01 were used as denoted in the text. ANOVA was used to determine the differences between the groups.

## Results

### Difference between surgeons

A one-way ANOVA was used to examine if DOT values were influenced by which surgeon was operating. Table 3 shows the difference among the means (M) and standard deviation (SD) of each of the four surgeons. The one-way ANOVA revealed no statistically significant difference between the DOT’s of each surgeon [*F* (3, 568) = 1.489, *p* > 0.05]. This indicated that no surgeon had a significant difference in their mean DOT compared to their colleagues. This assumption was to be established prior to analyzing the role of the nursing staff. The ANOVA results are presented in Table 4.

**Table 3:** Mean DOT between surgeons. Calculated mean of the difference of operative time (DOT) measured in minutes for individual surgeon along with the number of cases and the standard deviation (SD) between the DOT is represented

Surgeons	Number of Cases	Mean (Minutes)	SD
Surgeon A	88	1.95455	9.502461
Surgeon B	225	0.38222	9.493214
Surgeon C	161	2.19255	9.851723
Surgeon D	98	2.39796	12.155131

**Table 4:** One-way ANOVA DOT between surgeons. Calculated mean of the difference of operative time (DOT) measured in minutes for individual surgeon along with the *p*-value for the output of the one-way ANOVA analysis has been represented in the table. A one-way ANOVA compared the effect of individual surgeons on DOT and the analysis was not significant (*F* = 1.489, *p* = 0.217)

Covariate	Level	Mean	*p*-Value
Surgeon	Surgeon A	1.95455	0.217
	Surgeon B	0.38222	
	Surgeon C	2.19255	
	Surgeon D	2.39796	

### Primary test groups

A one-way ANOVA was used examine DOT in Group A and Group B cases. There was a significant difference between the DOT for Group A and Group B (*p* < 0.0001). Group B cases on average required less total operative time (M = − 2.75, SD = 9.24) than planned for. Group A cases required more total operative time (M = 4.34, SD = 9.37) than planned for. The mean values for each test group are illustrated in Table 5.

**Table 5:** One-way ANOVA DOT with or without ENT nurse. Calculated mean of the difference of operative time (DOT) and its corresponding 95% confidence interval (95% CI) measured in minutes for cases with and without ENT resource nurse participation, along with the *p*-value for the output of the one-way ANOVA analysis has been represented in the table. A one-way ANOVA compared the effect of ENT resource nurse participation during surgery on DOT and the analysis was significant (*F* = 4.493, *p* = 0.0000)

Covariate	Level	Mean (95% CI)	*p*-Value
DOT	Cases without an ENT Resource Nurse (Group A)	4.34 (3.33,5.35)	< 0.0001
	Cases with an ENT Resource Nurse (Group B)	−2.75 (−3.92,-1.59)	

A one-way ANOVA was also used to determine if the DOT was influenced by case length in isolation. There was no significant difference in DOT between cases greater than 30 min (Group II; M = 1.43, SD = 9.49) or lesser than 30 min (Group I; M = 0.24, SD = 9.42). Operative efficiency was not influenced by case length in isolation. These findings are presented in Table 6.

**Table 6:** One-way ANOVA - DOT and case length. Calculated mean of the difference of operative time (DOT) and its corresponding 95% confidence interval measured in minutes for cases with requested operating time lesser or greater 30 min, along with the *p*-value for the output of the one-way ANOVA analysis has been represented in the table. A one-way ANOVA compared the effect of case length on DOT and the analysis was not significant (*F* = 0.203, *p* = 0.064)

Covariate	Level	Mean (95% CI)	*p*-Value
DOT	Cases with ROT ≤30 min (Group I)	0.24 (−0.91,1.39)	0.0594
	Cases with ROT > 30 min (Group II)	1.43 (0.38,2.48)	

### Secondary test groups

A two-way ANOVA was then used to investigate if the length of the procedure and the presence of an ENT Resource Nurse influenced DOT values. The mean values suggest that procedures with either a ROT lesser than or greater than 30 min finished on average earlier when an ENT resource nurse is working. Table 7 shows means, 95% confidence interval and standard deviations among each of the four groups.

**Table 7:** Interactions between groups - Two-way ANOVA. Calculated mean of the difference of operative time (DOT) and its corresponding 95% confidence interval (95%CI) and standard deviation (SD) measured in minutes for cases with and without ENT resource nurse participation compared to cases with requested operating time lesser or greater 30 min, along with the *p*-value for the output of the two-way ANOVA analysis. A two-way ANOVA showed a statistically significant interaction between the effects of ENT resource nurse participation and length of surgery on DOT (*F* = 38.793, *p* = 0.0000)

Covariate	Level	Mean (95% CI)	SD	*p*-Value
ENT Nurse	Cases without an ENT Resource Nurse (Group A)	4.34 (3.33,5.35)	9.37	< 0.0001
	Cases with an ENT Resource Nurse (Group B)	−2.75 (− 3.92,-1.59)	9.24	
Case length	Cases with ROT ≤30 min (Group I)	0.24 (− 0.91,1.39)	9.42	0.0594
	Cases with ROT > 30 min (Group II)	1.43 (0.38,2.48)	9.49	
ENT Nurse* Case length	Group 1 - Group A*Group I	2.15 (0.64,3.66)	5.68	< 0.0001
	Group 2 - Group B*Group I	−1.19 (− 2.89,0.51)	5.01	
	Group 3 - Group A*Group II	6.53 (5.2,7.86)	11.85	
	Group 4 - Group B*Group II	−4.32 (− 5.93,-2.71)	10.85	

Post-hoc comparison using the Games-Howell procedure was conducted to determine which group means differed significantly. As the ROT increased, an ENT Resource nurse has a greater impact on reducing the DOT. In cases planned to last less than 30 min, ENT resource nurses reduced total operative time on average by more than 3 min (M = − 3.35, SD = 11.65). In cases planned to last more than 30 min, ENT resource nurses reduced total operative time on average by nearly 11 min (M = − 10.85, SD = 10.95). These results are presented in Table 8.

**Table 8:** Difference in means of interactions of ANOVA. Simple main effects analysis showed that means of interactions between Group 1 and Group 2; Group 1 and Group 4; Group 2 and Group 4; Group 3 and Group 4 were significantly different and more longer than interactions between Group 1 and Group 2; Group 2 and Group 3 which showed no differences between the groups. The mean difference is significant as denoted by * at level of α = 0.05 and ** at level of α = 0.01

	Group 1	Group 2	Group 3	Group 4
Group 1 (Group A*Group I)				
Group 2 (Group B*Group I)	−3.35**			
Group 3 (Group A*Group II)	4.38**	7.72**		
Group 4 (Group B*Group II)	−6.47**	−3.13*	−10.85**	

*Significant at level of α = 0.05 / **Significant at level of α = 0.01

**Fig. 2:**
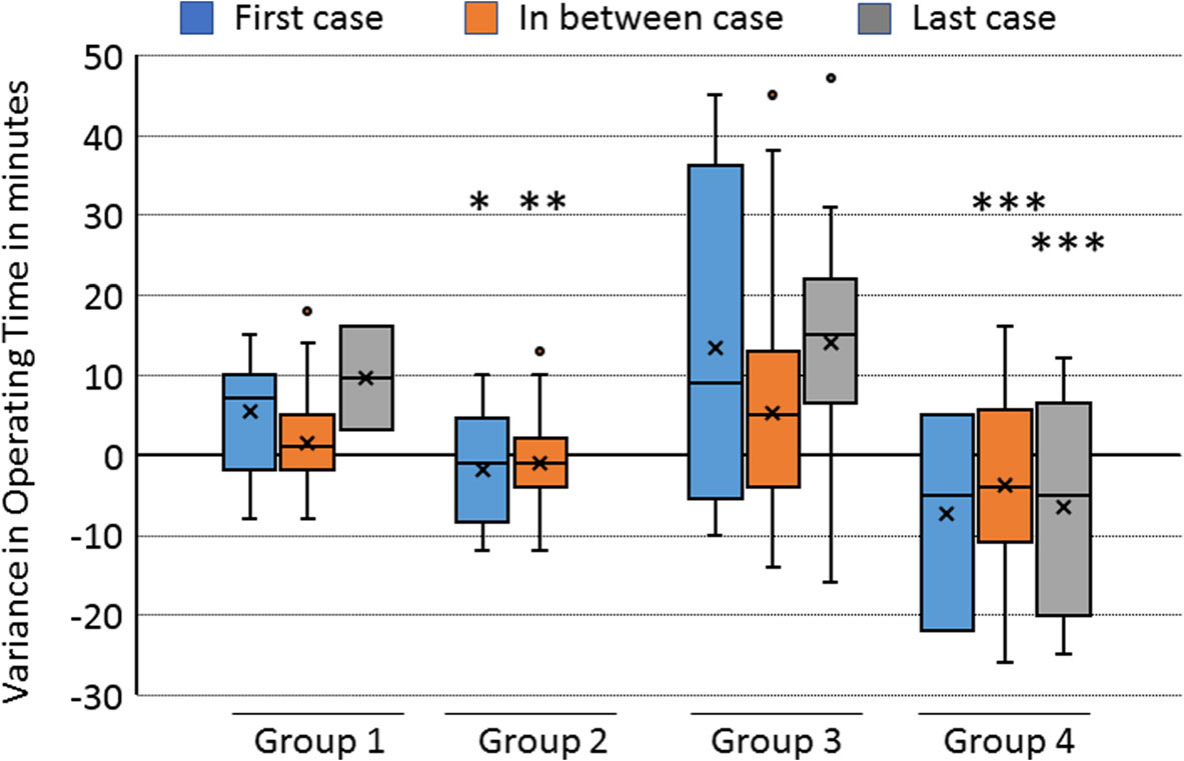
DOT variance due to case scheduling. Box Plot showing the difference of operative time (DOT) values among groups with and without ENT resource nurse participation and groups requiring < 30 or > 30 min ROT, which then were scheduled as first case (FC), in between cases (IBC) and last case (LC). Significant *p*-values (* = < 0.05; ** = < 0.001; *** = < 0.0001) were marked above respective box plots which were calculated using student t-test between the groups of cases scheduled as FC, IBC and LC respectively. ‘X’ in the box denotes the mean and the line dividing the box denotes median of the values

Further examination on the role of case scheduling on DOT values suggested that cases finish on average earlier when an ENT resource nurse is working regardless of case scheduling. As previously observed, when the ROT increases, the presence of an ENT Resource nurse has a significant impact on reducing operative time regardless of case scheduling with exception of the first case requiring > 30 mins ROT. The difference between DOT values in Group A and Group B cases requiring < 30 mins ROT and scheduled as first case (FC), in between cases (IBC) and last case (LC) was observed to be significant. With the exception of LC, significant *p*-values were observed in both FC and IBC (< 0.0011 and < 0.0003 repsectively). However, when the Group A and Group B cases requiring ROT > 30 mins were classified based on scheduele as FC, IBC and LC, highly significant *p*-values (both < 0.00001) were observed in both IBC and LC requiring ROT > 30 mins. The results have been summarized as a boxplot in the Fig. 2.

## Discussion

**Fig. 3:**
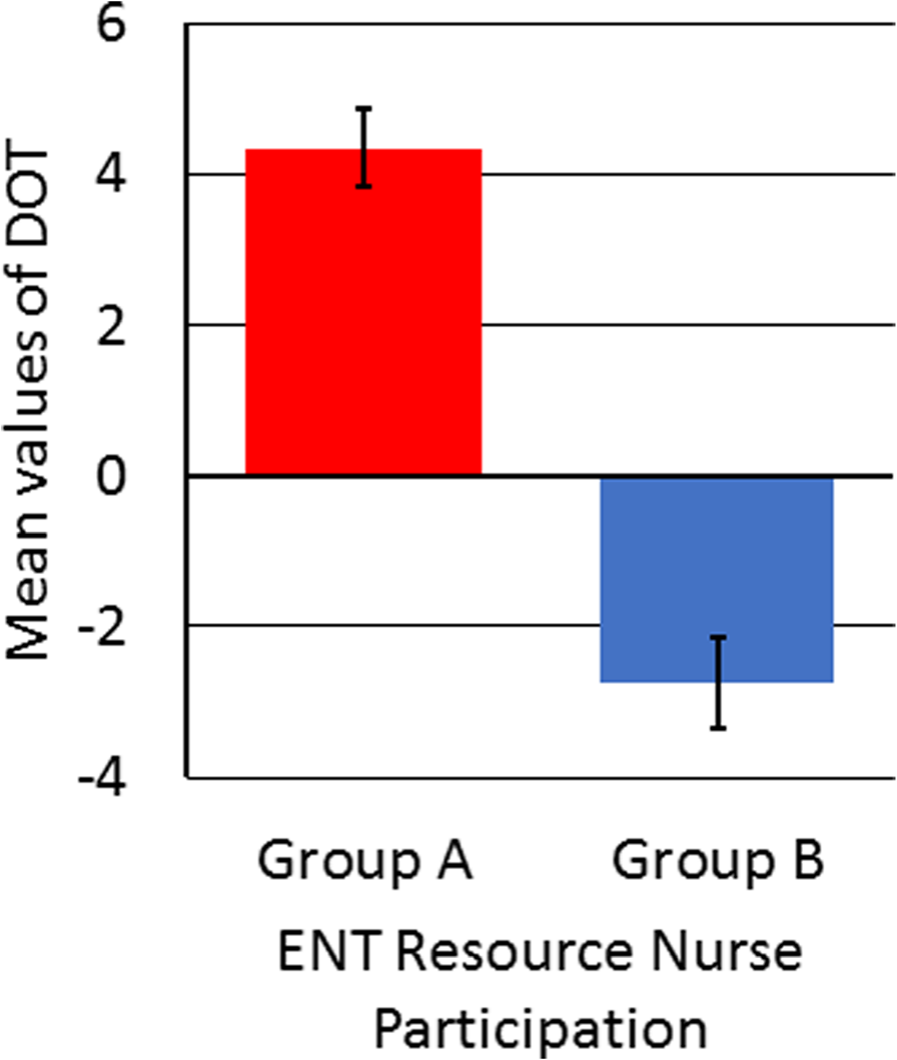
DOT variance due to ENT resources nurse participation. Bar graph representing the mean values of DOT among group with and without ENT resource nurse participation. Without an ENT nurse (Group A) cases finished on average 4.34 min late (M = 4.34, SE = 0.51). With an ENT nurse (Group B) cases finished on average 2.75 min early (M = − 2.75, SE = 0.59). Standard Error (SE) was derived by using the formula, standard deviation (SD)/square root of total number of samples (√n)

The results of our study demonstrate reduction in total operative time during common ENT surgeries, when specialized nursing staff are utilized. The results of the current study are in accordance with the findings of previous studies on the importance of specialization within the OR [9, 10]. Cases that were performed without the participation of a specialized ENT resource nurse, on average required more total operative time (Group A; M = 4.34, SD = 8.01) than planned for. On the contrary, cases that were performed with the participation of a specialized ENT resource nurse on average required less total operative time (Group B; M = − 2.75, SD = 10.81) than planned for. These results are represented in Fig. 3.

The data was then separated based on how long the ROT was. An ANOVA was first performed to compare DOT to ROT greater or less than 30 min. The ANOVA between DOT for long and short cases was not significantly influenced by case length (*p* > 0.01). Changes in efficiency occurred when the presence of an ENT resource nurse is factored into the analysis. Regardless of case length, presence of ENT resource nurses showed improvement in OR efficiency.

**Fig. 4:**
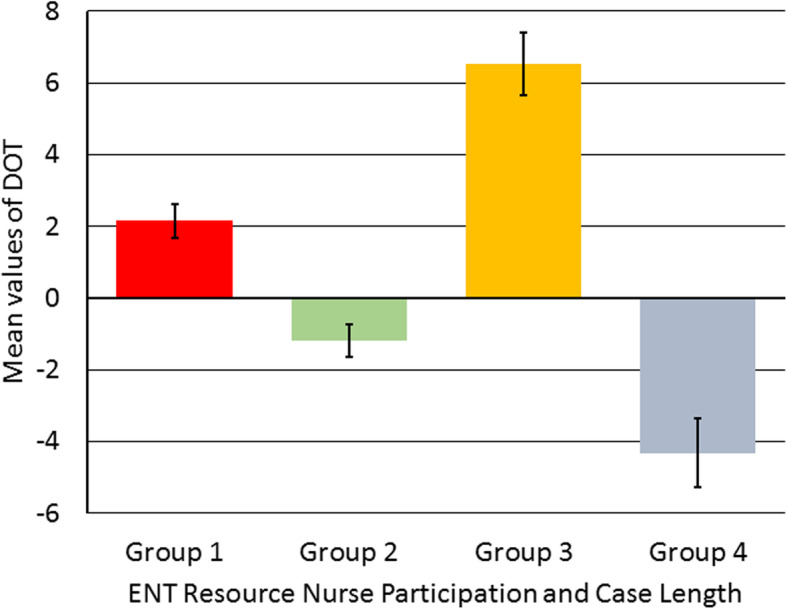
DOT variance due to case length and ENT resource nurse participation. Bar graph representing the mean values of DOT among groups with and without ENT resource nurse with different case length (ROT < 30 or > 30 min). In Group 1 without ENT nurse participation for cases with ROT < 30 min finished on average 2 min late (M = 2.15, SE = 0.47); Group 2 with ENT nurse participation for cases with ROT > 30 min finished on average 1 min early (M = -1.19, SE = 0.46); Group 3 without ENT nurse participation for cases with ROT > 30 min finished on average 7 min late (M = 6.53, SE = 0.86); Group 4 with ENT nurse participation for cases with ROT > 30 min finished on average 4 min early (M = -4.32, SE = 0.96). Standard Error (SE) was derived by using the formula, standard deviation (SD)/square root of total number of samples (√n)

Interestingly, cases that were greater than 30 min showed the largest increase in efficiency. With the presence of ENT resource nurse, cases under 30 min finished before the ROT. These cases finished on an average of 3.34 min earlier than those without an ENT resource nurse. With an ENT resource nurse presence, cases over 30 min also finished before the ROT. However, these cases finished on average 10.96 min earlier than those cases without an ENT resource nurse. The complexity and increased demands of longer cases are benefited to the most from the training of an ENT resource nurse. The results are illustrated in Fig. 4. In addition, scheduling does not the DOT among cases with ENT resource nurses (Fig. 2).

Compared to non-ENT nursing staff, specialized ENT resource nurses are more familiar with ENT procedures and better trained to setup/use ENT specific equipment. These factors have been identified as contributors to OR efficiency in prior studies [11]. Compared to non-ENT nursing staff, ENT resource nurses are likely to work on a higher proportion of ENT cases. This experience plus familiarity between OR staff has also been identified as a contributor to OR efficiency in prior research [11].

The current study has the strength from evaluating a large caseload from a single institution with consistent staffing. Using the ROT, allowed us to use a baseline comparison consistent to each of the four surgeons. Prior research has shown the value of using parameters similar to requested operative time within ENT for reasearch [12]. It must be identified that ROT has a direct relationship to DOT. The use of ROT in assessing OR efficiency is a novel concept specific to this study. It has not yet been validated as a measure of OR efficiency. We choose to use ROT and TOT as we felt that it eliminated differences in recording times for specific parameters such as skin-skin time, anesthesia time and turnover time. We hope to perform a future propsective study where we can ensure that these specific time parameters are recorded accurately and identically. The retrospective nature of the study also allowed for us to use surgical times that were objectively recorded without knowledge of this future study’s objective.

Cases with cofounding variables recorded on the OR report were removed (equipment issue, anesthesia delay, etc). However with this study being retrospective in nature, we were unable to identify confounding variables that may have occurred, but were not recorded at the time. While ROT allows to standardize the current analysis, it would be beneficial to review specific end points in the future. Skin-skin time, anesthesia time, and turnover time take up a large portion of TOT. However, these endpoints can be hard to validate retrospectively [13]. This was the reason why we chose to instead rely on the comparison between the ROT and TOT. A prospective study that can reliabily control the recording of additional endpoints would be recommended as follow up. Additionally, a comparison of inpatient and outpatient elective functional endoscopic sinus surgery (FESS) is planned for further assessment of efficiency improvement with ENT resource nurses. These studies may provide insight into exactly how a specialized ENT resource nurse improves OR efficiency.

## Conclusions

The current study results support the utilization of specialized ENT resource nurses. Their additional training results in decreased total operative time and improved OR efficiency. Procedural familiarity and equipment competence have been shown to improve OR efficiency [10, 11, 14]. Specialized ENT resource nurses help make the most of valuable OR time. Within the Saskatchewan Health Authority, we have demonstrated that specialized ENT nurses improve operating room efficiency for ENT cases. Cases that were anticipated to be longer than 30 min showed the greatest increase in efficiency when an ENT resource nurse was used. The highly specialized nature of ENT procedures and the variability between said procedures requires OR staff to be well trained. Operating room time is a valuable resource that should not be wasted. Speciality specific training for OR nursing staff can improve operating room efficiency within ENT.

## Data Availability

The data that support the findings of this study are available from the Saskatchewan Health Authority, but restrictions apply to the availability of this data, which was used under license for the current study, and so are not publicly available. Data is however available from the authors upon reasonable request and with permission of the Saskatchewan Health Authority.

## Abbreviations

OR: Operating Room
SHA: Saskatchewan Health Authority
ROT: Requested Operative Time (Predicted)
TOT: Total Operative Time (Actual)
DOT: Difference in Operative Time (ROT-TOT)
